# Autistic mothers’ perinatal well-being and parenting styles

**DOI:** 10.1177/13623613211065544

**Published:** 2022-02-01

**Authors:** Sarah Hampton, Carrie Allison, Ezra Aydin, Simon Baron-Cohen, Rosemary Holt

**Affiliations:** University of Cambridge, UK

**Keywords:** anxiety, autism, depression, parenting, perinatal, postnatal, pregnancy, satisfaction with life, stress

## Abstract

Autistic people may be at higher risk of perinatal mental health conditions, given that autism and mental health conditions commonly co-occur and that autistic people face additional stressors such as barriers to appropriate maternity care. This study explored self-reported stress, depression, anxiety and satisfaction with life during the third trimester of pregnancy (n = 27 autistic women; n = 25 non-autistic women), 2 to 3 months after birth (n = 24 autistic women; n = 26 non-autistic women) and 6 months after birth (n = 22 autistic women; n = 29 non-autistic women). Self-reported parenting confidence and parenting styles were explored at 6 months after birth. Autistic participants scored significantly higher than non-autistic participants on stress, depression and anxiety across the time-points as a whole, although there were no group differences for satisfaction with life. Anxiety scores significantly decreased over time for both groups. No group differences were found for parenting confidence nor parenting anxiety, nurturance, involvement or routine, although the autistic group scored lower on parenting discipline. The findings highlight the need for effective screening and support for perinatal mental health conditions for autistic people. Professionals working with autistic parents should be aware that autistic and non-autistic parents report being equally likely to engage in positive parenting behaviours such as nurturance and involvement.

**Lay abstract:**

Autistic people can have difficulties during pregnancy and after giving birth, such as difficulty getting health care that meets their needs. Autistic people may therefore have lower well-being than non-autistic people during this time. We asked autistic and non-autistic people to fill in questionnaires measuring stress, depression, anxiety and satisfaction with life. They were asked to do this once during pregnancy, once 2 to 3 months after giving birth and once 6 months after giving birth. At 6 months after giving birth, they also filled in questionnaires about parenting. The autistic parents had higher stress, depression and anxiety scores than the non-autistic parents. For both groups, scores for anxiety went down over time. There were no differences between the groups on satisfaction with their life or how confident they were as a parent. There were no differences between the groups on most areas of parenting style, although autistic parents scored lower on parenting discipline. This study suggests that autistic people may be more stressed, depressed and anxious than non-autistic people during pregnancy and after giving birth. Autistic people therefore need good quality support during this time. This study also suggests that autistic and non-autistic parents may be just as likely to parent in positive ways such as being sensitive to their baby’s needs.

## Background

Perinatal well-being^1^ is an important public health concern due to its impact on both mother and child. Perinatal anxiety, depression and stress have been associated with adverse birth outcomes (Accortt et al., 2015; Beydoun & Saftlas, 2008; Ding et al., 2014) as well as child developmental outcomes, including behavioural and emotional difficulties (Leis et al., 2014; Netsi et al., 2018; Prenoveau et al., 2017; Robinson et al., 2011). Symptoms of mental health conditions are common during the perinatal period. Recent estimates suggest that depressive symptoms have a prevalence of 17% during pregnancy and 13% during the first postnatal year (Underwood et al., 2016), and that anxiety symptoms have a prevalence of 18%–25% across pregnancy and 15% over the first 6 postnatal months (Dennis et al., 2017). Studies have tended to find a decrease in depression and anxiety from pregnancy to the postnatal period (Figueiredo & Conde, 2011; Heron et al., 2004) and an increase in satisfaction with life (Gebuza et al., 2014), suggesting that pregnancy may be a time of particular vulnerability (potentially due to alleviation of the physical challenges of pregnancy, and pregnancy-related anxieties concerning childbirth and the unborn child’s health becoming resolved after birth). These prevalence studies have tended to include participants from a range of socio-economic backgrounds, both primiparous and multiparous parents, and have involved predominantly western samples.

Little is known about the perinatal well-being of people with a diagnosis of autism, a condition characterised by differences in social interaction and communication, restricted and repetitive behaviours, and sensory processing differences (American Psychiatric Association (APA), 2013). Autistic people may be at increased risk of lower perinatal well-being given that a prior history of mental health conditions is a risk factor for poorer perinatal mental health (Lancaster et al., 2010) and that autistic groups report higher rates of mental health diagnoses, as opposed to typically developing populations (Lai et al., 2019). Autistic people may also face increased risk due to challenges such as heightened sensory sensitivities during pregnancy, which could relate to both increased sensitivity to the physical and biological changes associated with pregnancy, and to the environment (Gardner et al., 2016; Rogers et al., 2017; Talcer et al., 2021). Both could make it more difficult to access appropriate maternity care, for example, due to the sensory environment of health care facilities, and difficulties with touch during appointments and birth (Gardner et al., 2016; Rogers et al., 2017). Social and communication barriers to maternity care have also been identified for autistic people, including difficulty conveying needs and understanding information given during childbirth (Donovan, 2020), being less likely than non-autistic people to feel that the process of birth was explained to them (Pohl et al., 2020) and feeling judged by maternity care professionals (Gardner et al., 2016; Rogers et al., 2017). Such negative experiences could all increase the risk of poorer perinatal well-being. Indeed, one retrospective survey found that autistic mothers were more likely than non-autistic mothers to report having had prenatal and postnatal depression (Pohl et al., 2020). However, no research has explored mental health symptoms over the course of the perinatal period among autistic people.

Maternal mental health may impact parenting style and parenting confidence during the postnatal period. Associations have been found, for example, between maternal depression and anxiety symptomatology and lower parenting confidence (Kohlhoff & Barnett, 2013). Confidence concerning one’s parenting ability has implications for the well-being of both parent and child, including associations with parenting satisfaction (Elek et al., 2003) and child developmental outcomes (Coleman & Karraker, 2003). Furthermore, there is evidence that mothers with depression (Field, 2010; Stanley et al., 2004) and anxiety (Nicol-Harper et al., 2007) can show less sensitivity (the ability to correctly identify and respond to the infant’s cues and to provide appropriate warmth and acceptance), as measured during interactions with their infants. Sensitivity and other aspects of parenting style have been linked to child outcomes, with authoritative parenting (high sensitivity alongside an appropriate degree of control) being associated with more positive behavioural, cognitive and social outcomes than authoritarian (low sensitivity and high control) and permissive (high sensitivity and low control) styles (Rinaldi & Howe, 2012; Stright et al., 2008).

Research exploring autistic people’s postnatal parenting experiences is scarce. One survey study (Pohl et al., 2020) has explored autistic parenting beyond the postnatal period and found that autistic mothers reported greater difficulties than non-autistic mothers with aspects of parenting such as multitasking and domestic responsibilities. Autistic mothers were also more likely to report not coping, to find motherhood isolating, to feel judged and to feel unable to ask for support. There were no group differences, however, in prioritising their child’s needs above their own and seeking opportunities to boost their child’s confidence. This study is the first to quantitatively explore autistic people’s parenting experiences and styles during the postnatal period, a time window neglected in prior research. It is also the first to explore mental health symptoms over the course of the perinatal period among autistic parents, building upon prior work exploring retrospective reports of perinatal depression among autistic parents (Pohl et al., 2020).

This study explored autistic and non-autistic people’s self-reported anxiety, depression, stress and satisfaction with life during the third trimester of pregnancy, 2–3 months after birth and 6 months after birth. Parenting confidence and parenting styles were explored at 6 months after birth. It was hypothesised that autistic people may experience higher anxiety, depression and stress and lower satisfaction with life across the perinatal period, as well as lower parenting confidence.

## Method

### Participants

Participants completed questionnaires longitudinally, during the third trimester of pregnancy (n = 27 autistic women; n = 25 non-autistic women), 2–3 months after birth (n = 24 autistic women; n = 26 non-autistic women) and 6 months after birth (n = 22 autistic women; n = 29 non-autistic women). Twelve autistic participants and all non-autistic participants participated as part of a larger study exploring their child’s development (the Cambridge Human Imaging and Longitudinal Development (CHILD) study). The remainder were part of another study exploring autistic parents’ well-being (the Perinatal Experiences and Autism study). Participants were recruited through the ultrasound unit of the Rosie Maternity Hospital in Cambridge, the Cambridge Autism Research Database (CARD), autism-related and pregnancy-related support groups, social media and magazine advertisements. Those younger than 18 years were excluded as such parents may have different experiences relating to their younger age that might be difficult to tease apart from the experiences faced by autistic parents more broadly. Participants were not excluded due to pregnancy complications, given the rarity of the target sample and the need to maximise sample size. Ethics approval for the Perinatal Experiences and Autism study was obtained from the University of Cambridge Psychology Research Ethics Committee (PRE.2018.050). The CHILD study received NHS ethics approval (REC reference number: 12/EE/0393).

**Figure 1.**
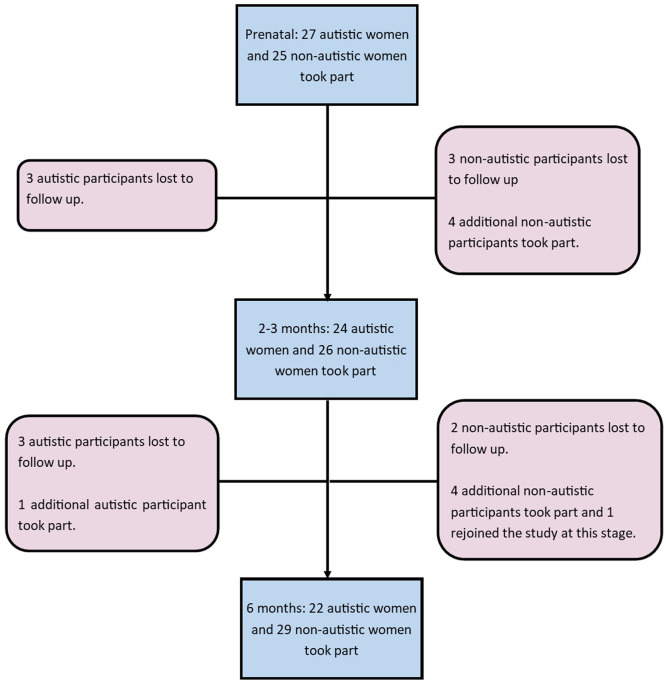
Participant attrition at each time-point.

Reasons for participant attrition are given in Figure 1. Participant attrition over time tended to be due to family commitments and scheduling difficulties. Participants who joined the study at later time-points predominantly did so due to ethics approval for the collection of questionnaire data within the CHILD study not yet having been granted at the time of their participation at earlier time-points. In addition, one autistic participant completed only the Cohen’s Perceived Stress Scale (CPSS) and State-Trait Anxiety Inventory (STAI) at 2–3 months. One autistic participant completed only the CPSS and STAI at 6 months. One non-autistic participant completed all but the Edinburgh Postnatal Depression Scale (EPDS) at 6 months.

Demographic information is presented in Table 1. Demographic questions and the Autism-Spectrum Quotient (AQ) were administered once during pregnancy. As such, changes across time-points (other than for age) are due to differing samples across time-points. The autistic group was significantly younger than the non-autistic group, had significantly lower education and income and was significantly more likely to have a diagnosis of a psychiatric condition. The autistic group had significantly fewer children (at the 2- to 3-month and 6-month time-points only) and was significantly more likely to reside in a country other than the United Kingdom (for the 2- to 3-month and 6-month time-points only). The autistic group scored significantly higher on the AQ than the non-autistic group. The groups did not significantly differ on the age or sex of their child, ethnicity, pregnancy conditions nor type of delivery. All infants were born at 36 weeks gestation or later. All participants were in a romantic partnership apart from two participants in the autistic group who took part at the prenatal time-point only. All participants identified as women.^2^ Four of the autistic group and none of the non-autistic group had an existing child with an autism diagnosis.

**Table 1.** Demographic information for the autistic and non-autistic groups at each time-point.

	Prenatal			2–3 months			6 months		
	Autistic (n = 27)	Non-autistic (n = 25)	p-value	Autistic (n = 24)	Non-autistic (n = 26)	p-value	Autistic (n = 22)	Non-autistic (n = 29)	p-value
Mother’s mean age (SD)^a^	30.84 (4.05)	33.84 (2.69)	**0.003**	31.04 (4.07)	34.33 (2.75)	**0.002**	31.48 (3.07)	34.89 (3.48)	**0.001**
Mean age of child in weeks/gestational weeks (SD)^a^	32.01 (2.58)	31.22 (2.26)	0.25	11.05 (1.67)	10.78 (1.58)	0.56	27.42 (1.41)	26.70 (1.01)	0.05
Sex of child (female: male)^b^	13:13	11:14	0.78	12:12	11:15	0.78	10:12	14:15	1
Ethnicity^b^			0.13			0.05			0.07
White	25 (93%)	19 (76%)		24 (100%)	21 (81%)		21 (100%)	24 (83%)	
Non-White	2 (7%)	6 (24%)		0 (0%)	5 (19%)		0 (0%)	5 (17%)	
Educational level^b^			**0.004**			**0.01**			**0.004**
Undergraduate or above	15 (56%)	23 (92%)		14 (58%)	24 (92%)		12 (57%)	27 (93%)	
A level or below	12 (44%)	2 (8%)		10 (42%)	2 (8%)		9 (43%)	2 (7%)	
Annual household income (£)^b^			**<0.001**			**<0.001**			**0.001**
>50,000	8 (31%)	20 (80%)		8 (33%)	22 (85%)		7 (33%)	23 (79%)	
⩽50,000	18 (69%)	5 (20%)		16 (67%)	4 (15%)		14 (67%)	6 (21%)	
Psychiatric conditions^b^			**<0.001**			**<0.001**			**<0.001**
None	8 (30%)	23 (92%)		8 (33%)	24 (92%)		7 (33%)	26 (90%)	
Depression	2 (7%)	1 (4%)		2 (8%)	1 (4%)		1 (5%)	2 (7%)	
Depression and anxiety	9 (33%)	1 (4%)		6 (25%)	1 (4%)		6 (29%)	1 (3%)	
OCD and anxiety	2 (7%)	0 (0%)		2 (8%)	0 (0%)		2 (9%)	0 (0%)	
Other	6 (22%)	0 (0%)		6 (25%)	0 (0%)		5 (24%)	0 (0%)	
Country of residence^b^			0.08			**0.02**			**0.004**
UK	21 (77%)	25 (100%)		18 (75%)	26 (100%)		16 (73%)	29 (100%)	
USA	5 (19%)	0 (0%)		5 (21%)	0 (0%)		4 (18%)	0 (0%)	
Ireland	1 (4%)	0 (0%)		1 (4%)	0 (0%)		2 (9%)	0 (0%)	
Number of children (not including current pregnancy)^b^			0.14			**0.02**			**0.02**
0	21 (77%)	16 (64%)		19 (79%)	15 (58%)		17 (77%)	17 (59%)	
1	2 (7%)	7 (28%)		1 (4%)	9 (35%)		1 (5%)	10 (34%)	
2	4 (15%)	2 (8%)		4 (17%)	2 (8%)		4 (18%)	2 (7%)	
Pregnancy conditions^b^			0.33			–			–
Gestational diabetes	4 (15%)	1 (4%)		–	–		–	–	
Polyhydramnios	1 (4%)	1 (4%)		–	–		–	–	
Pre-eclampsia	0 (0%)	1 (4%)		–	–		–	–	
Type of delivery^b^			–			0.68			0.45
Vaginal	–	–		11 (46%)	15 (58%)		9 (41%)	17 (59%)	
Assisted vaginal (forceps or ventouse)	–	–		3 (12%)	3 (11%)		3 (14%)	3 (10%)	
Caesarean section	–	–		10 (42%)	8 (31%)		10 (45%)	9 (31%)	
Mean AQ score (SD)^a^	39.56 (5.39)	14.40 (7.46)	**<0.001**	39.58 (5.69)	15.69 (7.86)	**<0.001**	40.76 (4.59)	15.62 (7.39)	**<0.001**

Information unavailable for one autistic participant who took part at the prenatal time-point only for mother’s age, sex of child and income. Information unavailable for one autistic participant who took part at the 6-month time-point only for mother’s age, ethnicity, education, income and psychiatric conditions. OCD: obsessive-compulsive disorder. Boldface values are significant at *p* < 0.05.

a T-test performed.

b Fisher’s exact test performed.

### Procedure

Questionnaires were completed either in person, via post or online through email or Qualtrics. Participants gave written informed consent. The CPSS, STAI, EPDS and Satisfaction with Life Scale (SWLS) were completed at all three time-points. The Karitane Parenting Confidence Scale (KPCS) and Infancy Parenting Styles Questionnaire (IPSQ) were completed at 6 months only. The AQ was completed during pregnancy to gain an indication of the participants’ levels of autistic traits.

#### The AQ

The AQ (Baron-Cohen et al., 2001) is a self-report measure of autistic traits. Scores range from 0 to 50, with higher scores indicating greater autistic traits and a score of 32 or above indicating potentially clinically significant levels of autistic traits. The AQ has good reliability (Baron-Cohen et al., 2001) and has good sensitivity (0.88) though lower specificity (0.20; Ashwood et al., 2016).

#### CPSS

The CPSS (Cohen et al., 1983) is a self-report questionnaire measuring stress. Respondents are asked to report on their feelings and thoughts during the last month, such as ‘In the last month, how often have you found that you could not cope with all the things that you had to do?’. Scores range from 0 to 40, with higher scores indicating higher stress. A cut-off score of 20 or more indicates high stress. The CPSS has a Cronbach’s alpha between 0.84 and 0.86 (Cohen et al., 1983) and has been widely used in both pregnant and postnatal populations.

#### STAI

The STAI (Spielberger et al., 1983) is a self-report questionnaire measuring anxiety. To minimise participant burden, participants completed only the state anxiety subscale, which measures feelings in the current moment, such as ‘I am tense’ and ‘I am worried’. Scores range from 20 to 80, with higher scores indicating greater anxiety. A cut-off score of 40 is commonly used to indicate potential clinical levels of anxiety. The STAI has good validity and internal consistency in pregnant and postnatal populations (Meades & Ayers, 2011).

#### EPDS

The EPDS (Cox et al., 1987) is a self-report questionnaire measuring depression. Respondents are asked to report on their feelings in the past 7 days, such as ‘In the past 7 days, I have felt sad or miserable’. Scores range from 0 to 30 and higher scores indicate greater depressive symptoms, with scores of 13 or above indicating the presence of a depressive illness. The EPDS is commonly used as a screening tool for perinatal depression and has good reliability and validity (Bergink et al., 2011; Eberhard-Gran et al., 2001).

#### SWLS

The SWLS (Diener et al., 1985) is a self-report measure of satisfaction with life. Respondents are asked to report on their satisfaction with their life as a whole, such as ‘In most ways my life is close to my ideal’. Scores range from 5 to 35, with higher scores indicating greater satisfaction. The scale has good reliability and validity (Pavot & Diener, 2008), including during the perinatal period (Aasheim et al., 2014).

#### KPCS

The KPCS (Črnčec et al., 2008) is a self-report questionnaire measuring parenting confidence in parents of children aged 0–12 months. Respondents are asked to report on how they generally feel, such as ‘I feel I am doing a good job as a mother/father’. Scores range from 0 to 45, with higher scores indicating greater confidence. A cut-off score of 39 or below indicates clinically low parenting confidence (36–39 = ‘mild clinical range’, 31–35 = ‘moderate clinical range’, 30 or less = ‘severe clinical range’). The KPCS has a Cronbach’s alpha of 0.81 and test–retest reliability of 0.88 (Črnčec et al., 2008).

#### IPSQ

The IPSQ (Arnott & Brown, 2013) is a self-report questionnaire measuring parenting styles in parents of children aged 0–12 months. The IPSQ consists of five subscales: ‘discipline’ (belief that an infant can be naughty and need to control the infant’s behaviour, such as ‘It is never too young to start disciplining a child’), ‘routine’ (encouraging sleep and feeding routines, such as ‘A routine makes a baby calm and secure’), ‘anxiety’ (anxiety about the infant’s health or development, such as ‘I worry a lot about my baby’), ‘nurturance’ (responding sensitively to the infant, such as ‘I generally like to keep my baby as close as possible to me’) and ‘involvement’ (promoting the infant’s development, such as ‘I encourage my baby to develop skills such as walking or talking’). Discipline and routine are intended to correspond to the dimension of control, and nurturance to the dimension of warmth, in relation to models of parenting styles for older children (Baumrind, 1978). Cronbach’s alpha for the subscales range from 0.65 to 0.88 (Arnott & Brown, 2013).

### Community involvement

Feedback on a draft of the manuscript was given by email by two autistic mothers who did not take part in the research. They were contacted through the Autism Centre of Excellence Advisory Panel (a database of autistic people volunteering to advise on research). The mothers felt that the language used was appropriate and gave suggestions relating to the interpretations of results (particularly around parenting style) which were then implemented. While this feedback was useful in helping to ensure that the language used and interpretation of results was more acceptable to the autistic community, it is acknowledged that feedback from only two mothers is unlikely to represent the diversity of perspectives present in the autistic community.

### Data analysis

The Research Electronic Data Capture platform (Harris et al., 2009, 2019) was used to record data. Prenatally, data were missing for one item on the CPSS for one autistic participant and for one item on the EPDS for one autistic participant. These values were imputed using the individual participant’s mode for that questionnaire. Data for income were unavailable for one autistic participant who took part at the prenatal time-point only and one autistic participant who took part at the 6-month time-point only. Data for psychiatric conditions were unavailable for one autistic participant who took part at the 6-month time-point only. Values were imputed with the mode of the autistic group. Analyses were run with and without the imputed data and the pattern of results did not change.

For analyses involving one time-point only, linear regressions were conducted with group (autistic/non-autistic) as a predictor of scores. Parity (primiparous or multiparous) and income were included as covariates. A history of mental health conditions is associated with poorer perinatal mental health (Lancaster et al., 2010). As such, whether or not participants had received a prior diagnosis of depression was included as a covariate in the analysis of depression scores. Similarly, a prior diagnosis of an anxiety disorder (as classified according to the *Diagnostic and Statistical Manual of Mental Disorders* (5th ed.; *DSM*-5; APA, 2013)) was included as a covariate in the analysis of anxiety scores.

To provide descriptive statistics on how prior and current mental health compare, for both depression and anxiety, the percentage of participants in each group with a prior diagnosis is reported alongside the percentage of participants scoring above the cut-off at each time-point. The percentage of participants scoring in the clinical range is reported for stress and parenting confidence. For stress, depression and anxiety, the percentage of participants scoring above the cut-off during pregnancy who do not score above the cut-off at either postnatal time-point are reported in the text, in addition to the percentage scoring above the cut-off during at least one postnatal time-point who had not scored above the cut-off during pregnancy. These data are presented to provide an indication of the timing of onset of clinical levels of symptoms.

For those questionnaires completed at all time-points, multilevel models (using maximum likelihood estimation) were conducted. Group, time-point, an interaction between group and time-point, income and parity were included as fixed effects, with scores on the questionnaires as the outcome. For each model, a random intercept for participant was included and models additionally involving a random slope for time-point (to allow for the effect of time-point to differ across participants) and models involving a random slope for time-point and a first-order autoregressive covariance structure (as time-points were approximately evenly spaced) were also considered. The inclusion of random slopes and covariance structures did not significantly improve the models and resulted in higher Akaike information criterion (AIC)/Bayesian information criterion (BIC) values, and therefore random slopes and covariance structures were not included in the final models.

For models where the assumptions of normality of residuals and/or homoscedasticity were violated, robust standard errors and p-values were calculated through bootstrapping with 2000 replications.

Participant attrition may influence results pertaining to changes over time, for example, whether those experiencing greater challenges feel unable to continue their participation. Analyses were run with and without those with incomplete data for one or more time-points and results relating to time-point did not substantially differ (full results are provided in Supplementary Tables S1 to S4). As such, only analyses involving all participants (including those with incomplete data) are reported.

## Results

### Stress

**Figure 2.**
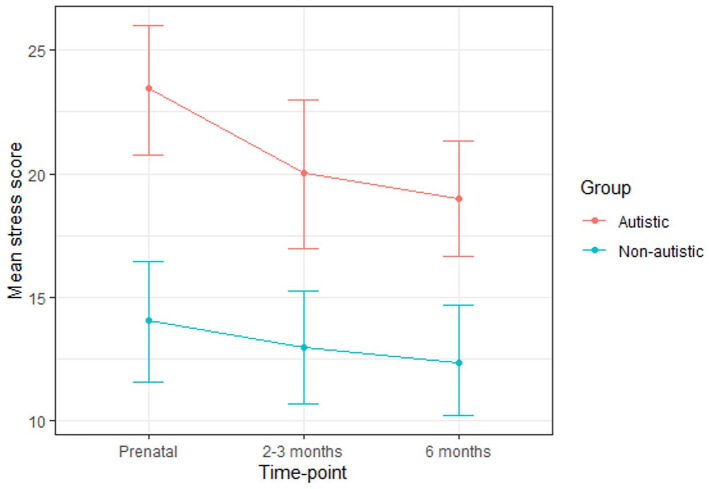
Mean stress scores for the autistic and non-autistic groups at each time-point (error bars represent 95% confidence intervals).

At each time-point, the autistic group had higher stress scores than the non-autistic group and a greater percentage scored above the cut-off (Table 2; Figure 2). For the autistic group, a minority of those who scored above the cut-off during pregnancy did not go on to score above the cut-off at either postnatal time-point (29%) and, similarly, only a minority of those who scored above the cut-off during at least one postnatal time-point had not scored above the cut-off during pregnancy (13%). Conversely, for the non-autistic group, all of those who scored above the cut-off during pregnancy did not go on to score above the cut-off at either postnatal time-point and all of those who scored above the cut-off during at least one postnatal time-point had not scored above the cut-off during pregnancy.

**Table 2.** Stress scores and the number and percentage of participants scoring above the cut-off at each time-point.

	Prenatal		2–3 months		6 months	
	Autistic (n = 27)	Non-autistic (n = 25)	Autistic (n = 24)	Non-autistic (n = 26)	Autistic (n = 22)	Non-autistic (n = 29)
Mean stress score (SD)	23.48 (7.17)	14.08 (6.26)	20.04 (7.78)	13.00 (6.15)	19.00 (5.82)	12.35 (6.17)
N (%) above cut-off (⩾20)	20 (74)	4 (16)	14 (58)	3 (12)	11 (50)	4 (14)

A multilevel model revealed that group significantly predicted stress scores (Table 3). Post hoc tests (with Tukey adjustment) confirmed that the autistic group scored significantly higher at all time-points (prenatal: B (SE) = 7.62 (1.98), p = 0.004; 2–3 months: B (SE) = 6.50 (2.02), p = 0.02; 6 months: B (SE) = 6.28 (1.99), p = 0.03). Time-point did not significantly predict stress scores and there was no significant group-by-time-point interaction.

**Table 3.** Results of the model for stress scores.

	B (SE)	p-value
Group	7.50 (1.84)	**<0.001**
Time-point	−1.00 (0.66)	0.12
Group × Time-Point	−0.74 (0.91)	0.43
Income	−3.35 (1.67)	0.05
Parity	1.76 (1.55)	0.26

Boldface values are significant at *p* < 0.05.

### Depression

**Figure 3.**
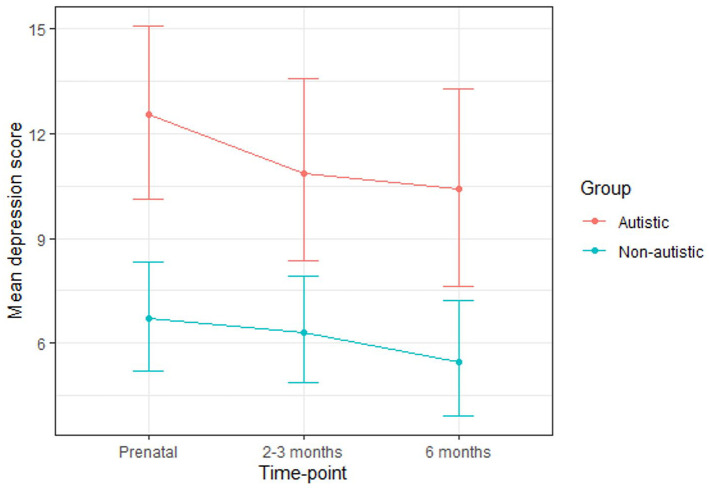
Mean depression scores for the autistic and non-autistic groups at each time-point (error bars represent 95% confidence intervals).

The autistic group had higher depression scores than the non-autistic group at each time-point (Table 4; Figure 3). A greater percentage of the autistic group than the non-autistic group scored above the cut-off for depression at each time-point and a greater percentage had a prior diagnosis of depression. For each time-point, the percentage of those scoring above the cut-off and the percentage of those with a prior diagnosis of depression were similar. Many (autistic group: 43%; non-autistic group: 67%) of those who scored above the cut-off during pregnancy did not go on to score above the cut-off at either postnatal time-point. Around half (autistic group: 54%; non-autistic group: 50%) of those who scored above the cut-off during at least one postnatal time-point did not score above the cut-off during pregnancy.

**Table 4.** Depression scores, prior depression diagnosis and the number and percentage of participants scoring above the cut-off at each time-point.

	Prenatal		2–3 months		6 months	
	Autistic (n = 27)	Non-autistic (n = 25)	Autistic (n = 23)	Non-autistic (n = 26)	Autistic (n = 21)	Non-autistic (n = 28)
Mean depression score (SD)	12.56 (6.74)	6.72 (4.20)	10.87 (6.43)	6.31 (4.01)	10.43 (6.26)	5.46 (4.58)
N (%) above cut-off (⩾13)	13 (48)	3 (12)	11 (48)	2 (8)	8 (38)	3 (11)
N (%) with a prior depression diagnosis	14 (52)	2 (8)	11 (48)	2 (8)	9 (43)	3 (11)

A multilevel model revealed that group significantly predicted depression scores, indicating that the autistic group scored significantly higher across the three time-points as a whole (Table 5). Post hoc tests (with Tukey adjustment) indicated that group differences did not reach significance at any particular time-point (prenatal: B (SE) = 3.77 (1.74), p = 0.27; 2–3 months: B (SE) = 3.81 (1.76), p = 0.27; 6 months: B (SE) = 4.19 (1.75), p = 0.17). Time-point did not significantly predict depression scores and there was no significant group-by-time-point interaction.

**Table 5.** Results of the model for depression scores.

	B (SE)	p-value
Group	3.72 (1.69)	**0.03**
Time-point	−0.70 (0.50)	0.18
Group × Time-Point	0.19 (0.71)	0.79
Income	−0.75 (1.44)	0.60
Parity	−0.07 (1.33)	0.94
Diagnosis of depression	3.80 (1.51)	**0.01**

Bootstrapped standard errors and p-values reported.

Boldface values are significant at *p* < 0.05.

### Anxiety

**Figure 4.**
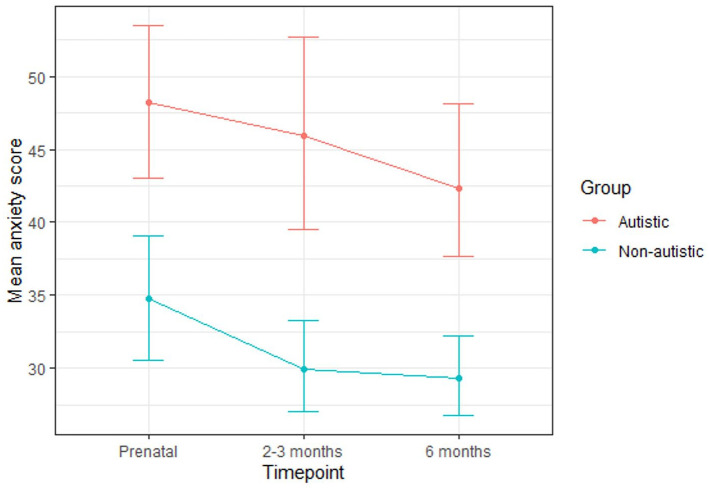
Mean anxiety scores for the autistic and non-autistic groups at each time-point (error bars represent 95% confidence intervals).

The autistic group had higher anxiety scores than the non-autistic group at each time-point (Table 6; Figure 4). A greater percentage of the autistic group than the non-autistic group scored above the cut-off for anxiety at each time-point and had a prior diagnosis of an anxiety disorder. For both groups, a greater percentage scored above the cut-off at the prenatal and 2-3 month time-points than had a prior diagnosis of an anxiety disorder. For the non-autistic group, but not the autistic group, this was also the case at the 6-month time-point. Many (autistic group: 40%; non-autistic group: 40%) of those who scored above the cut-off during pregnancy did not go on to score above the cut-off at either postnatal time-point. The percentage of those who scored above the cut-off during at least one postnatal time-point having not scored above the cut-off during pregnancy was greater for the non-autistic group (40%) than the autistic group (20%).

**Table 6.** Anxiety scores, prior anxiety diagnosis and the number and percentage of participants scoring above the cut-off at each time-point.

	Prenatal		2–3 months		6 months	
	Autistic (n = 27)	Non-autistic (n = 25)	Autistic (n = 23)	Non-autistic (n = 26)	Autistic (n = 22)	Non-autistic (n = 29)
Mean anxiety score (SD)	48.22 (13.72)	34.76 (11.46)	45.96 (16.01)	29.96 (8.65)	42.36 (12.67)	29.34 (7.61)
N (%) above cut-off (⩾40)	21 (78)	7 (28)	13 (54)	5 (19)	10 (45)	3 (10)
N (%) with a prior anxiety disorder diagnosis	14 (52)	1 (4)	11 (48)	1 (4)	10 (45)	1 (3)

A multilevel model revealed that group significantly predicted anxiety scores (Table 7). Post hoc tests (with Tukey adjustment) indicated that the autistic group scored significantly higher at the 2- to 3-month (B (SE) = 13.78 (4.07), p = 0.02) and 6-month (B (SE) = 12.35 (4.02), p = 0.04) time-points but not the prenatal time-point (B (SE) = 9.25 (4.05), p = 0.22). Time-point significantly predicted anxiety scores. Post hoc tests (with Tukey adjustment) revealed a significant decrease from the prenatal to the 6-month time-point (B (SE) = −6.34 (2.39), p = 0.02), although there was no significant difference between the prenatal and 2- to 3-month time-points (B (SE) = −5.45 (2.40), p = 0.06), nor between the 2- to 3-month and 6-month time-points (B (SE) = −0.89 (2.32), p = 0.92). There was no significant group-by-time-point interaction.

**Table 7.** Results of the model for anxiety scores.

	B (SE)	p-value
Group	10.18 (4.00)	**0.01**
Time-point	−3.11 (1.23)	**0.01**
Group × Time-Point	1.50 (1.74)	0.37
Income	−3.29 (3.13)	0.29
Parity	0.81 (2.95)	0.80
Diagnosis of anxiety	3.63 (3.65)	0.32

Bootstrapped standard errors and p-values reported.

Boldface values are significant at *p* < 0.05.

### Satisfaction with life

**Figure 5.**
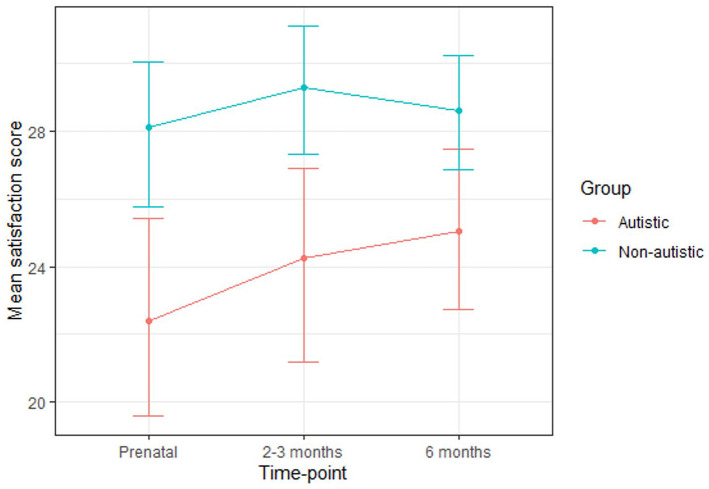
Mean satisfaction with life scores for the autistic and non-autistic groups at each time-point (error bars represent 95% confidence intervals).

While the autistic group scored lower than the non-autistic group on satisfaction with life at each time-point (Table 8; Figure 5), a multilevel model revealed that neither group nor time-point significantly predicted scores and there was no significant group-by-time-point interaction (Table 9).

**Table 8.** Satisfaction with life scores at each time-point.

	Prenatal		2–3 months		6 months	
	Autistic (n = 27)	Non-autistic (n = 25)	Autistic (n = 23)	Non-autistic (n = 26)	Autistic (n = 21)	Non-autistic (n = 29)
Mean satisfaction with life score (SD)	22.41 (7.86)	28.12 (5.75)	24.26 (7.12)	29.31 (5.07)	25.05 (5.69)	28.62 (4.82)

**Table 9.** Results of the model for satisfaction with life scores.

	B (SE)	p-value
Group	−2.81 (1.83)	0.13
Time-point	0.53 (0.51)	0.28
Group × Time-Point	0.44 (0.74)	0.56
Income	4.56 (1.68)	**0.004**
Parity	−0.37 (1.53)	0.84

Bootstrapped standard errors and p-values reported.

Boldface values are significant at *p* < 0.05.

### Infancy parenting styles

Linear regressions revealed that the autistic group scored significantly lower than the non-autistic group on the discipline subscale of the IPSQ, although there were no significant group differences on anxiety, involvement, nurturance or routine (Table 10).

**Table 10.** Scores and results of regression models for the IPSQ subscales.

	Autistic (n = 22)	Non-autistic (n = 29)	B (SE)	p-value
Mean anxiety (SD)	11.10 (3.27)	9.28 (2.62)		
Group			1.12 (0.93)	0.24
Income			−0.57 (0.92)	0.54
Parity			−2.34 (0.84)	**0.01**
Model: F(3, 46) = 4.42, p = 0.01, R^2^ = 0.22				
Mean discipline (SD)^a^	9.71 (3.44)	11.93 (4.94)		
Group			−3.53 (1.73)	**0.04**
Income			−1.93 (1.84)	0.30
Parity			−1.90 (1.31)	0.18
Model: F(3, 46) = 2.26, p = 0.09, R^2^ = 0.13				
Mean involvement (SD)	17.91 (3.00)	18.76 (2.57)		
Group			−1.35 (0.93)	0.16
Income			−0.56 (0.92)	0.55
Parity			−1.21 (0.84)	0.16
Model: F(3, 46) = 1.16, p = 0.33, R^2^ = 0.07				
Mean nurturance (SD)	14.48 (2.89)	12.76 (2.08)		
Group			1.38 (0.84)	0.11
Income			−0.69 (0.83)	0.41
Parity			0.08 (0.76)	0.92
Model: F(3, 46) = 2.18, p = 0.10, R^2^ = 0.12				
Mean routine (SD)	15.86 (4.66)	16.90 (3.36)		
Group			−0.49 (1.36)	0.72
Income			0.78 (1.34)	0.56
Parity			0.89 (1.22)	0.47
Model: F(3, 46) = 0.54, p = 0.66, R^2^ = 0.03				

IPSQ: Infancy Parenting Styles Questionnaire.

Boldface values are significant at *p* < 0.05.

a Bootstrapped standard errors and p-values reported.

### Parenting confidence

A slightly greater percentage of the autistic group than the non-autistic group scored within the moderate and severe clinical ranges for low parenting confidence (Table 11), although linear regression revealed no significant association between group and KPCS scores (Table 12).

**Table 11.** KPCS scores and the number and percentage of participants scoring in the clinical range.

	Autistic (n = 22)	Non-autistic (n = 29)
Mean KPCS score (SD)	37.62 (5.95)	40.08 (3.33)
N (%) below clinical cut-off (⩽39)	10 (48%)	8 (28%)
N (%) Mild clinical range (36–39)	4 (19%)	7 (24%)
N (%) Moderate clinical range (31–35)	3 (14%)	1 (3%)
N (%) Severe clinical range (⩽30)	3 (14%)	0 (0%)

KPCS: Karitane Parenting Confidence Scale.

**Table 12.** Results of the regression model for KPCS scores.

	B (SE)	p-value
Group	−1.97 (1.45)	0.18
Income	1.69 (1.51)	0.25
Parity	2.17 (1.16)	0.07
Model: F(3, 46) = 3.16, p = 0.03, R^2^ = 0.17		

KPCS: Karitane Parenting Confidence Scale.

Bootstrapped standard errors and p-values reported.

## Discussion

This is the first study to explore trajectories of perinatal well-being among autistic people. The findings indicate higher perinatal stress, depression and anxiety symptoms among autistic people. This is consistent with evidence that autistic people have increased risk of mental health conditions (Lai et al., 2019), including prenatal and postnatal depression (Pohl et al., 2020).

Higher perinatal stress, depression and anxiety may in part be due to the perinatal challenges that some autistic people face, including heightened sensory experiences, a lack of autism understanding among health care professionals, communication barriers to healthcare (Gardner et al., 2016; Rogers et al., 2017) and parenting challenges (Pohl et al., 2020). Hormonal differences may also play a role, given findings of altered hormone levels and increased risk of hormone-related conditions among autistic females (Gasser et al., 2020; Pohl et al., 2014). For anxiety, group differences reached significance for the postnatal time-points only, indicating that postnatal stressors may be particularly influential in driving group differences. These stressors may include parenting challenges such as multitasking, as well as feeling isolated, judged and unsupported (Pohl et al., 2020). The groups did not significantly differ on satisfaction with life scores. While the measures of stress, depression and anxiety focus on recent feelings, the SWLS concerns satisfaction with one’s life as a whole and as such may be less sensitive to current changes in well-being.

It is possible that group differences in well-being are not particular to the perinatal period but reflect lower pre-existing well-being among autistic people (indeed, a greater proportion of the autistic group had a prior diagnosis of a psychiatric condition). However, group differences in depression and anxiety scores remained after accounting for a prior diagnosis of depression or anxiety, respectively, suggesting that higher depression and anxiety among the autistic group may not solely be the result of baseline differences in mental health. The possibility nevertheless remains that group differences in stress, depression and anxiety are, at least in part, driven by higher pre-existing levels of mental health symptomatology (including obsessive-compulsive disorder (OCD) and other mental health conditions) among autistic participants. It is important that maternity services are aware that autistic people may be vulnerable to worse perinatal mental health and this vulnerability may be linked to higher pre-existing mental health symptomatology among autistic people. Further research should attempt to elucidate the mechanisms that contribute towards worse perinatal mental health among some autistic people, including the role of pre-existing vulnerability to mental health conditions.

For both groups, the percentage of participants with a prior diagnosis of depression and those scoring in the clinical range for depression were similar. However, the percentage of participants scoring in the clinical range for anxiety tended to be greater than the percentage with a prior diagnosis. This may suggest that the majority of cases of perinatal depression are preceded by a diagnosis of depression, whereas a substantial percentage of cases of perinatal anxiety may not be preceded by an anxiety disorder diagnosis. This may be due to new cases of anxiety arising during the perinatal period or may reflect under-diagnosis of anxiety pre-pregnancy. This may have clinical implications, such that while maternity services may be able to consider a prior diagnosis of depression as a vulnerability factor for depression, a prior diagnosis of an anxiety disorder may be a less strong predictor of perinatal anxiety. Perinatal anxiety may therefore be harder to detect and this may lead to parents not getting the support they require.

Stress, anxiety and depression scores tended to decrease over time for both groups, although this decrease only reached significance for anxiety. This pattern reflects prior findings in the general population of higher depression and anxiety during pregnancy than postnatally (Figueiredo & Conde, 2011; Heron et al., 2004). Improved well-being postnatally may be due to pregnancy-related worries (concerning childbirth and the unborn child’s health) becoming resolved after birth. It may also be due to physiological factors such as changes in hormone levels or the alleviation of the physical burden of pregnancy. The presence of social and financial support from others (such as a partner, family, friends and a peer support network of other parents) may also be a protective factor against poorer postnatal well-being (Pao et al., 2019). It would be important for future research to consider the role that a support network plays in this regard for autistic people, particularly given associations between autism and increased loneliness (Ee et al. 2019), which could lead to greater feelings of isolation postnatally and represent a risk factor for worse well-being. For both groups, many of those scoring above the cut-off on the questionnaires during pregnancy no longer scored above the cut-off postnatally, while many of those scoring above the cut-off postnatally had not previously scored above the cut-off during pregnancy. This suggests substantial movement across the thresholds for stress, depression and anxiety symptoms over the course of the perinatal period and echoes similar findings for depression (Underwood et al., 2016). For stress in particular, there was greater movement across the threshold over time for the non-autistic group than the autistic group, perhaps reflecting greater pre-existing vulnerability to stress within the autistic group rather than fluctuations specific to the perinatal period.

The autistic group scored lower on the discipline subscale of the IPSQ, indicating that this group were less willing to endorse beliefs such as ‘My baby sometimes does things that are naughty’ and ‘It is never too young to start disciplining a child’. It is important to note that this subscale concerns infants below 1 year old and may not reflect parents’ attitude towards providing appropriate discipline with older offspring. The group difference in discipline may be due to autistic participants taking a more accepting approach towards their infant’s behaviour, rather than judging their behaviour negatively. A reduced tendency towards conformity among autistic people (Yafai et al., 2014) may enable a more accepting approach towards parenting that is less constrained by dominant ideas of what constitutes acceptable child behaviour. While potentially bringing benefits, such an approach could also make certain aspects of parenting more challenging. The concept of parenthood is informed by culturally variable social norms and expectations (such as gendered role distributions, for example). If autistic people approach parenting in ways that are less constrained by these expectations, this could influence the feelings of being judged and misunderstood by others that autistic mothers report experiencing (Pohl et al., 2020).

The groups did not differ in their self-perception of their parenting anxiety, involvement, nurturance or routine. This suggests that autistic and non-autistic people may be just as likely to demonstrate these parenting behaviours. The groups did not significantly differ on parenting confidence, although a greater percentage of autistic than non-autistic participants scored in the clinical range. This may indicate that there is a slightly greater proportion of autistic parents who would benefit from support to improve their parenting confidence. The group difference in those scoring in the clinical range is small, however, limiting the ability to make strong conclusions based on this finding. The lack of group differences in most areas of parenting is in contrast to the presence of group differences in mental health. Given associations between mental health and parenting confidence (Kohlhoff & Barnett, 2013) and sensitivity (Field, 2010), it may be expected that higher mental health symptoms among the autistic group would accompany lower confidence, involvement and nurturance. The lack of group differences in parenting may be influenced by the small sample size, the self-report nature of the data or may indicate that autistic parents are able to compensate for higher mental health symptoms. It is possible, for example, that autistic mothers may have increased awareness of the challenges they face as a parent due to having received an autism diagnosis and may therefore attempt to compensate for these challenges to a greater extent than non-autistic mothers.

### Limitations

This data set is novel as it follows autistic people longitudinally through pregnancy and the postnatal period. However, due to recruitment challenges, the sample size is small. Currently pregnant autistic people are a rare group, given that females are diagnosed with autism less frequently than males and that those recruited were required to be in a narrow window of pregnancy to take part. The longitudinal nature of the study imposed a further burden upon participants, which may be prohibitive for those navigating the challenges of pregnancy and postnatal responsibilities. Some null findings may be due to a lack of power and future studies employing larger samples are necessary to corroborate the present findings.

Questionnaires were administered at one prenatal time-point only meaning that changes in well-being across pregnancy (perhaps due to changing concerns and physical experiences as pregnancy progresses) were not explored.

The autistic and non-autistic groups were not well matched. The groups differed on socio-economic factors, prior history of psychiatric conditions and country of residence, all of which may affect experiences of perinatal health care. Future studies would benefit from the inclusion of a well-matched comparison group.

Stress, depression and anxiety scores prior to pregnancy were not collected and it is therefore unclear whether the perinatal period represents a particularly vulnerable time for lower well-being among autistic people or rather that group differences reflect pre-existing differences in mental health. Prospective studies exploring well-being from before pregnancy until the postnatal period could tease apart these issues.

## Conclusions and clinical implications

Autistic people may be vulnerable to higher perinatal stress, depression and anxiety than non-autistic people and perinatal healthcare professionals should be aware of this increased vulnerability. The findings highlight the need for effective screening and support surrounding perinatal well-being for autistic people. Given the variation among participants’ scores, it is likely that different levels of support are required for different individuals and professionals should provide personalised support according to the challenges the individual is facing. The tentative finding of an improvement in well-being from pregnancy to the postnatal period may indicate that, for both autistic and non-autistic people, pregnancy may be a period of increased need for support. Some autistic parents may benefit from support to improve their parenting confidence. However, professionals working with autistic parents should be aware that autistic people report being no less likely to engage in positive parenting behaviours such as providing nurturance, involvement and routine.

## Supplemental Material

sj-docx-1-aut-10.1177_13623613211065544 – Supplemental material for Autistic mothers’ perinatal well-being and parenting styles Supplemental material, sj-docx-1-aut-10.1177_13623613211065544 for Autistic mothers’ perinatal well-being and parenting styles by Sarah Hampton, Carrie Allison, Ezra Aydin, Simon Baron-Cohen and Rosemary Holt in Autism

## Notes

1. The term *well-being* is used throughout as a broad term encompassing aspects of mental health, including, but not limited to, stress, depression, anxiety and satisfaction with life.

2. While all participants identified as women, it is acknowledged that autistic people may have higher rates of gender-diverse identities (Warrier et al., 2020) and that gender expression and norms may be perceived differently by autistic women (Kanfiszer et al., 2017). This may affect how pregnancy and the role of being a mother are experienced for autistic people.
