# Supplementary material for: Autistic mothers’ perinatal well-being and parenting styles
Source: Autism. 2022 Feb 1;26(7):1805–20. doi: 10.1177/13623613211065544 (PMC9483197; doi:10.1177/13623613211065544)
Supplement: sj-docx-1-aut-10.1177_13623613211065544 – Supplemental material for Autistic mothers’ perinatal well-being and parenting styles [file sj-docx-1-aut-10.1177_13623613211065544.docx]

Table S1 Results of sensitivity analysis on stress scores involving only participants with complete data for all time-points

|  | B (SE) | p-value |
| --- | --- | --- |
| Group | 6.44 (2.08) | **0.004** |
| Time-point | -0.95 (0.71) | 0.19 |
| Group*Time-point | -0.43 (0.99) | 0.67 |
| Income | -3.06 (1.77) | 0.09 |
| Parity | 1.31 (1.74) | 0.46 |
| Post-hoc group comparisons |  |  |
| Prenatal | -6.44 (2.17) | 0.05 |
| 2-3 months | -6.02 (2.17) | 0.08 |
| 6 months | -5.57 (2.17) | 0.13 |

Table S2 Results of sensitivity analysis on depression scores involving only participants with complete data for all time-points

|  | B (SE) | p-value |
| --- | --- | --- |
| Group | 2.45 (2.09) | 0.24 |
| Time-point | -0.74 (0.57) | 0.21 |
| Group*Time-point | 0.39 (0.79) | 0.66 |
| Income | -0.88 (1.75) | 0.61 |
| Parity | -0.56 (1.72) | 0.72 |
| Depression diagnosis | 3.04 (1.80) | 0.08 |

Table S3 Results of sensitivity analysis on anxiety scores involving only participants with complete data for all time-points

|  | B (SE) | p-value |
| --- | --- | --- |
| Group | 9.12 (4.47) | **0.04** |
| Time-point | -3.50 (1.38) | **0.01** |
| Group*Time-point | 2.60 (1.93) | 0.19 |
| Income | -2.14 (3.50) | 0.51 |
| Parity | 1.43 (3.44) | 0.68 |
| Anxiety diagnosis | 1.29 (3.95) | 0.77 |
| Post-hoc group comparisons |  |  |
| Prenatal | -8.61 (4.49) | 0.41 |
| 2-3 months | -12.75 (4.49) | 0.07 |
| 6 months | -13.80 (4.49) | **0.04** |
| Post-hoc time-point comparisons |  |  |
| Prenatal – 2–3 months | -4.90 (2.87) | 0.10 |
| 2-3 months – 6 months | -2.10 (2.15) | 0.34 |
| Prenatal – 6 months | -7.00 (3.22) | **0.04** |

Table S4 Results of sensitivity analysis on satisfaction with life scores involving only participants with complete data for all time-points

|  | B (SE) | p-value |
| --- | --- | --- |
| Group | -3.31 (2.07) | 0.11 |
| Time-point | 0.63 (0.55) | 0.27 |
| Group*Time-point | 0.20 (0.78) | 0.82 |
| Income | 4.41 (1.88) | **0.03** |
| Parity | -0.93 (1.72) | 0.62 |
